# Specific immunotherapy by the sublingual route for respiratory allergy

**DOI:** 10.1186/1710-1492-6-29

**Published:** 2010-11-09

**Authors:** Cristoforo Incorvaia, Simonetta Masieri, Patrizia Berto, Silvia Scurati, Franco Frati

**Affiliations:** 1 Allergy/Pulmonary rehabilitation, Istituti Clinici di Perfezionamento, Milan, Italy; 2 ENT Clinic, University La Sapienza, Rome, Italy; 3 PBE Consulting, Verona, Italy; 4 Medical and Scientific Department, Stallergenes, Milan, Italy

## Abstract

Specific immunotherapy is the only treatment able to act on the causes and not only on the symptoms of respiratory allergy. Sublingual immunotherapy (SLIT) was introduced as an option to subcutaneous immunotherapy (SCIT), the clinical effectiveness of which is partly counterbalanced by the issue of adverse systemic reactions, which occur at a frequency of about 0.2% of injections and 2-5% of the patients and may also be life-threatening. A large number of trials, globally evaluated by several meta-analyses, demonstrated that SLIT is an effective and safe treatment for allergic rhinitis and allergic asthma, severe reactions being extremely rare. The application of SLIT is favored by a good compliance, higher than that reported for SCIT, in which the injections are a major factor for noncompliance because of inconvenience, and by its cost-effectiveness. In fact, a number of studies showed that SLIT may be very beneficial to the healthcare system, especially when its effectiveness persists after treatment withdrawal because of the induced immunologic changes.

## Introduction

Allergic diseases have high and increasing world prevalence [1,2]. In particular, respiratory allergy is caused by sensitization to environmental aeroallergens such as pollens, house dust mites, moulds, and animal epithelia and is clinically expressed as rhinitis and asthma.

The management of respiratory allergy relies upon, when possible, allergen avoidance, drug treatment, and allergen-specific immunotherapy (SIT) [3]. SIT is the practice of administering gradually increasing doses of the specific causative allergen to reduce the clinical reactivity of allergic subjects. SIT has central importance because of its ability to modify the natural history of the disease and to extend its effectiveness also after treatment withdrawal, provided it is administered for an adequate duration [4]. The subcutaneous route has been for decades the traditional route of administration, but in recent years the sublingual route emerged as an actual treatment option [5]. The main reason to introduce sublingual immunotherapy (SLIT) was the safety problems with subcutaneous immunotherapy (SCIT), which may include systemic reactions, sometimes severe and, though very rarely, even fatal [6].

The first studies on SLIT used low allergen dosages [7,8] but it was soon apparent that doses much higher than those administered by SCIT were needed to ensure clinical efficacy. In consensus documents, an optimal dosage as high as at least 50 times the dose administered by injection was suggested [3], though this ratio may be not pertinent for all products.

The high number of trials on SLIT conducted in recent years allowed an accurate evidence-based assessment of its effectiveness by several meta-analyses on the whole patient population as well as on subgroups defined by clinical expression (rhinitis and asthma) or age (adults and children) parameters [9-16]. At the same time, other studies evaluated important aspects defining the suitability of SLIT, such as the compliance and the cost-effectiveness.

This review is aimed at providing to the reader a comprehensive view of this treatment that is proposed for very common diseases.

## Effectiveness of Slit

The clinical efficacy of SLIT, as of SIT in general, is evaluated by the decrease in symptom scores of rhinitis and asthma and in consumption of symptomatic drugs. Many placebo-controlled studies are conducted on small patient populations and cannot achieve reliable statistical significance, but their combined evaluation by the tool of meta-analysis is considered an adequate method to obtain more solid data. The results are expressed as standardized mean difference (SMD) and allow to compare the effect of SLIT on actively and placebo-treated patients.

The first meta-analysis on SLIT was published in 2005, including 22 randomized controlled studies [9]. Such analysis demonstrated a significantly higher efficacy of SLIT versus placebo, with SMD corresponding to -0.42 for symptom scores (p = 0.002) and to -0.43 for medication scores (p = 0.00003). The authors did not succeed in detecting differences concerning subgroups, such as the kind of allergen and patients age, because of the relatively low numbers.

The meta-analysis by Olaguibel et al dealt with 7 randomized controlled studies conducted on children aged up to 14 years [10] and found that SLIT was significantly effective on asthma symptoms (SMD -1.42; p = 0.01) and on drug consumption (SMD -1.01; p = 0.06), while the improvement did not reach the significance for nasal and conjunctival symptoms.

A further meta-analysis on SLIT in children was published in 2006 [11]. In this case the evaluation concerned the efficacy on allergic rhinitis, including 10 randomized controlled studies with an overall number of 484 patients (245 actively and 239 placebo-treated). A significant reduction of both symptoms (SMD -0.56, p = 0.02) and medication (SMD -0.76, p = 0.03) was observed. A remarkable aspect was provided from the sub-analysis addressing the length of treatment and the kind of allergen administered, which demonstrated a higher efficacy for durations longer than 18 months and for pollen allergens compared to house dust mites. In the same year appeared a meta-analysis on the efficacy of SLIT in asthma, which included 25 studies with an overall number of 1706 patients [12]. Calculating the SMD, the reduction of asthmatic symptoms did not reach the statistical significance but using the intention-to-treat method for outcome measures, significant decreases of asthma symptoms and drug consumption and significant improvement of lung function and bronchial hyperreactivity were detected. The number needed to treat (NNT) to avoid leaving 1 patient with the same symptoms or worse was 3.7, that is, in the range of those reported for injective SIT in asthmatic and rhinitic patients. Better results were obtained by analyzing 9 studies on pediatric patients: the total number of patients was 441, 232 actively treated and 209 placebo-treated patients [13]. A significant reduction was found in both symptoms scores (SMD -1.14; p = 0.02) and drug use (SMD -1.63, p = 0.007).

The limit of meta-analysis is the relevant heterogeneity of the included studies, mainly because of different scoring systems. Recent evaluations considered altogether the meta-analyses but reached contrasting conclusions. According to Nieto et al, who checked the data reported in the original studies, the meta-analyses show "discrepancies, inconsistencies, and lack of robustness" and "do not provide enough evidence" for the current routine use of SLIT in patients with allergic asthma or rhinoconjunctivitis [14]. By contrast, the overall evaluation of all meta-analyses (5 on SLIT and 2 on SCIT) by Compalati et al, despite a significant heterogeneity of studies and 1 negative meta-analysis, lead the authors to conclude that "SIT can be recommended for the treatment of respiratory allergy because of its efficacy in reducing asthma and rhinitis symptoms" [15]. However, the major effects on asthma were achieved using the subcutaneous route.

The latest meta-analysis was limited to studies on grass pollen allergy and it was found that SLIT significantly reduces both symptoms (SMD -0.32) and medication use (SMD -0.33) compared with placebo, is more efficacious in adults than in children, and. prolonging the duration of preseasonal treatment for more than 12 weeks improves the treatment efficacy [16].

A data source alternative to meta-analyses are studies conducted on large numbers of patients that allow adequate statistical power. The recent preparations for SLIT in tablets of grass pollen extract were evaluated on large populations, including 855 adults treated by a single grass (*Phleum pratense*) extract [17], 628 adults treated using a 5-grass pollen extract [18], and 278 children treated using the latter preparation [19]. These studies showed a highly significant improvement in symptoms and rescue-medications scores in actively treated compared with placebo-treated patients during the grass pollen season. Nevertheless, there is yet criticism on the therapeutic role of SLIT, especially regarding the efficacy in children, in asthmatic patients and in house dust mite sensitization [20]. A balanced and updated review by Larenas-Linnemann concluded that additional studies are needed to improve the knowledge on the long-term effects and preventive action of SLIT, as well as on the optimal dosing for dust mites [21]. This is confirmed by a recent meta-analysis on SLIT with mite extracts, showing, as stated by the authors, "promising evidence of efficacy" but suggesting "more data, derived from large population-based high quality studies" [22]. Concerning pollen allergy, a number of dosing regimens have been studied, which include preseasonal, co-seasonal, and pre-co-seasonal protocols, but the optimal regimen and dose has only been established for a few allergenes [23]. Focusing SLIT safety and tolerability, all systematic reviews and meta-analyses found that the most common adverse events are local reactions in the oropharynx and that systemic reactions such as asthma, rhinitis, or urticaria are quite rare [24,25]. In the study by Calamita et al [12] was calculated the number needed to harm, that is, the number of patients to treat to have 1 adverse reaction, which corresponded to 14.3. Anaphylactic reactions are extremely rare, but an increased risk is apparent in subjects undergoing SLIT because of previous systemic reactions to SCIT [25,26], especially when no updosing regimens are used [25].

## Compliance with SLIT

The first studies on compliance with SIT were conducted when only the subcutaneous route was available and reported low compliance, ranging from 45% to 60%, but the demanding schedules used, based on very frequent injections, accounted for this outcome, as shown by the patients' recognizing of inconvenience of the doctor's visit to receive the injections as the major cause of noncompliance [27]. More recent studies using less aggressive schedules reported a better compliance, estimated at 75% to 90%, inconvenience remaining the major cause of noncompliance, followed by the cost of the treatment [28].

SLIT has different compliance issues than SCIT, because it is administered at home by patients themselves, and thus it is not affected by most causes reported for noncompliance to allergen injections, having instead compliance problems similar to drug treatment. Some studies not specifically designed for compliance (for instance safety and tolerability analyses) reported that treatment withdrawal is frequently caused by repeated local reactions in the mouth or at the gastrointestinal level [9,29]. Moreover, as previously noted in SCIT studies, it was observed that a lack of compliance to SLIT may be caused by the erroneous perception that once allergic symptoms are improved, SLIT is no more needed [30]. Concerning specific compliance and adherence studies, the available data indicate quite satisfactory results. In a study on 319 patients mainly addressing the efficacy of SLIT, the adherence to treatment (assessed by measuring the consumed allergen extracts) was estimated to be good, i.e. > 80%, in 72% of the patients, and fair, i.e. > 60%, in 18% of the patients [31]. The approach of evaluating the adherence by measuring the consume of allergen extracts was used in other 3 studies, which were facilitated by the fact that preparations in tablets or in liquid monodoses were employed. In the first of these, adult patients under SLIT treatment were asked by phone (without being warned in advance) to count the remaining tablets in the box; the phone interview was done during the first year in patients treated for dust mite allergy and during the first pollen season in patients treated for pollen allergy; the adherence value comprised between 75% and 97% [32]. The other 2 surveys were real-life studies investigating the compliance to SLIT in 443 adult and adolescent patients [33] and in 71 children [34], respectively. In the study on adults, the data on compliance were obtained (by unscheduled phone calls) after 3 months from all the patients, while after 6 months the data were obtained from 266 patients because the remaining 217 patients had received preseasonal SLIT of 3-4 months duration; 76.3% of the patients after 3 months and 74.8% of the patients after 6 months had compliance higher than 90%; in 4% of the patients SLIT was discontinued because of various reasons unrelated to the treatment and in 1% of the patients because of side effects possibly related to treatment [33]. Similarly, in the study on children with the same product in monodoses, parents were interviewed by unscheduled phone calls at the third and sixth month of SLIT and asked to count at once the remaining doses; a compliance rate higher than 75% was found in 85% of the children at the third month and in 84% of the children at the sixth month and the major cause of withdrawal (5.6% of the cases) was the cost of the treatment, while side effects accounted for 1.4% of stopping [34].

In a study comparing compliance to SLIT, SCIT and local nasal immunotherapy (LNIT) in children, the data on SLIT concerned 806 patients, 173 of whom (21.4%) were noncompliant, with a highly significant difference (p < 0.0001) for better compliance in hospital setting (90.5%) compared to private office setting (61.2%); the most common reason of withdrawal was the cost of the treatment, reported globally in 36.4% of the patients, compared with 39.6% of the patients undergoing SCIT and 13.3% of the patiens undergoing LNIT, followed by inconvenience (17.9% of the patients vs. 24.2% of the patients urdergoing SCIT and 3.5% of the patients undergoing LNIT), feeling of inefficacy (24.9% of the patients vs. 13% of the patients undergoing SCIT and 18.3% of the patients undergoing LNIT), and side effects (5.8% of the patients vs. 8.7% of the patients undergoing SCIT and 56.6% of the patients undergoing LNIT) [35].

In a survey on the allergist's opinion about the factors positively influencing the adherence to SLIT, the issues judged most important were the patient's perception of efficacy, reimbursability, tolerability, and the patient's education [36]. Actually, this latest aspect is likely to be of pivotal importance, as showed in a recent study reporting a clear difference in compliance between the patients receiving a complete educational course on SLIT and those receiving only standard instructions [37].

## Cost Effectiveness of SLIT

Currently, cost-effectiveness is a very important issue in medicine and some studies actually addressed the economic aspects of SLIT. The first published study dealt with the evaluation of the cost effectiveness of SLIT in children with allergic rhinitis and asthma, assessed by direct costs (drugs, specialists visits, and SLIT) and indirect costs (costs resulting from children school and parental work loss) indicating that high-dose SLIT may be effective in reducing the global cost of allergic rhinitis and asthma [38].

An overall number of 135 patients were analyzed, 46 with perennial and 89 with seasonal allergy, with comparable gender and age distribution. A substantial reduction was found in all outcome measures during SLIT compared with the previous period. The average annual cost/patient was € 2672 before SLIT initiation and € 629/year during SLIT. Similar results were found for allergen subgroups. The asthma analysis involved 41 children undergoing SLIT and 35 controls and also showed a substantial reduction in outcomes, though the direct cost per patient over the 4 years follow-up was € 1182 for SLIT-treated children and € 1100 for controls. These findings showed that high-dose SLIT may be effective in reducing the global cost of allergic rhinitis and asthma and comparably expensive to conventional drug treatment in children with allergic asthma over a 4-year follow-up.

The second study was addressed on a cohort of adults with pollen allergy and was conducted using a decision tree developed and populated with epidemiologic and resource utilization data concerning about 2200 patients [39]. The target population were young adults suffering from pollen-induced rhinitis with or without asthma. The time perspective was established at 6 years in order to include the long term effectiveness of SLIT, and the patients' data were collected in 25 Italian centers.

The main assessment criteria for the 2 different strategies were: a) costs, including the direct medical costs assessed in the National Health Service (NHS) perspective (visits, diagnostic procedures, drugs, SLIT and hospitalizations) and the direct plus indirect costs (lost working days) and patient out-of-pocket expenses assessed in the societal perspective; b) effectiveness end-point, including the number of patients improved and number of asthma cases avoided; and c) incremental cost per improved patient and incremental cost per asthma case avoided.

Cost analysis was performed in order to define the lowest cost strategy. A mean cost per patient treated over a period of 6 years was calculated for each therapeutic strategy and for each of the 2 perspectives studied. The SLIT strategy resulted in less expense in terms of both direct and indirect costs. The break-even point of SLIT, that is, the time in which the overall cost of treatment for SLIT patients becomes lower than that for patients receiving only drugs for the societal perspective was reached at year 4.

The third study evaluated the economics of SLIT in patients with pollen allergy and allergic rhinitis alone or associated with asthma compared with standard case controls [40]. This study was made by a longitudinal observational database operated by a network of allergy centers. The patients were randomly assigned to SLIT (plus drugs as needed) or to treatment using drugs alone. The outcome measures included the use of: drugs, SLIT, visits, and tests. The costs were assessed in the perspective of the Italian NHS.

The results showed that the overall per patient yearly cost of treatment was higher in SLIT patients, both in the whole sample (€ 311 vs. € 180/patient) and in subgroups of patients with rhinitis (€ 288 vs. € 116) and rhinitis associated with asthma (€ 362 vs. € 230). Patients with rhinitis plus asthma generated more costs than those with rhinitis alone in both groups. Nevertheless, considerable savings were obtained in the cost of symptomatic drugs (-22% for rhinitis and -34% for rhinitis plus asthma) in SLIT patients, thus focusing the use of symptomatic drugs as an important indicator of effective allergy control.

A recent study evaluated for the first time the cost-effectiveness of SLIT in patients with mite-induced asthma, reporting a higher mean annual cost in the first year in subjects treated with SLIT plus the needed symptomatic drugs compared with subjects only receiving drug treatment, while an economic advantage was apparent in the ensuing years and especially when SLIT was discontinued 3 three years because of the persistent good clinical control in SLIT-treated patients [41].

Two other studies concerned the evaluation of economic aspects of SLIT performed using oral tablets for grass pollen allergen in northern [42] and southern Europe [43]. The aim of the first study was the assessment of cost-effectiveness of grass allergen tablets compared with the use of symptomatic drugs in 7 northern European countries. A societal perspective was adopted, and the analysis had a 9-year time horizon. The main outcome measure was quality adjusted life years (QALYs). QALYs measure the patients' health-related quality of life (QoL) on a scale from 0 (death) to 1 (perfect health), and is a multi-attribute utility scale that can generate a single numeric index of health-related QoL.

The findings of this study showed that the grass allergen tablet was clinically superior to symptomatic treatment, producing statistically significant differences for all efficacy end-points, including the number of QALYs gained (0.976 vs. 0.947). There was a significantly higher usage of rescue medications (antihistamines and corticosteroids), and more hours missed from work (productivity losses) in the symptomatic treatment group. The cost per QALY gained in the grass allergen tablet group was similar in the 7 countries (€ 12930 to € 18263 for an annual cost of the grass allergen tablet of € 1500). This pharmacoeconomic analysis confirmed that SLIT is a cost-effective intervention for the prevention of grass pollen induced rhinoconjunctivitis in northern European countries with a tablet price below € 6. For example, in Germany the price of the tablet is € 2.95, corresponding to a yearly treatment cost of € 358, according to a 9-year time horizon.

The second study [42] assessed the cost-effectiveness of grass pollen oral tablets in patients with grass pollen-induced rhinitis living in 4 southern European countries (Spain, France, Italy, and Austria). A prospective pharmacoeconomic analysis was carried out alongside a multinational clinical trial measuring the efficacy of grass pollen tablets. Pooled data on resource use and health outcomes were collected. A societal perspective was adopted, and the analysis had a 9-year time horizon. The primary outcome measure was QALYs. SLIT was superior to standard care for all efficacy endpoints, including QALYs gained, and resulted in significantly less use of rescue medication and fewer hours missed from work. The oral grass allergen tablet was cost-effective for all countries for an annual price in the range of € 1500-1900.

Concerning the cost comparison between SCIT and SLIT, the first study was conducted in France in 2007. The cost/efficacy analysis was performed using a decision tree model by the perspective of the French Social Security, comparing IT and current symptomatic treatment in adults and children with dust mite and pollen allergy [44]. In adults, the savings with SCIT were € 393 for dust mite and € 1327 for pollen allergy over a 6-year period. In children, the savings were € 583 for dust mite and € 597 for pollen allergy over a 7-year period. The data from SLIT showed, as expected because of no need of visits for injections, higher savings, corresponding to € 3158 for dust mite and € 1708 for pollen allergy in adults and to € 3938 for dust mite and € 824 for pollen allergy in children. A recent study was done in Czech Republic on patients with allergic rhinoconjunctivitis receiving SLIT, SCIT, or only drugs for 3 years [45]. The total average direct cost per patient was € 416 for SLIT and € 482 for SCIT. A SLIT-treated patient paid less than a SCIT-treated patient for all out-of-pocket costs (€ 176 vs. € 255) but paid more for allergen extracts (€ 72 vs. € 55). The sum of direct and indirect costs gave, over the 3-year treatment, € 684 for SLIT and € 1004 for SCIT. These findings clearly indicate that SLIT, and SCIT as well, may be very beneficial to the healthcare system, in that either it could bring more clinical effectiveness at a reduced cost versus standard drug treatment or it could bring extra benefit at an acceptable extra cost, thus fully accomplishing the significance of the term cost-effectiveness [46]. Still, considering the growing expectations of prescribers and payers alongside with the spreading use of new technologies, further studies are needed to account for the different types of immunotherapy, patients, management processes and healthcare settings.

## Conclusion

SLIT has gained ample evidence of efficacy and safety and in some European countries is currently more frequently used than SCIT. Apart the better safety, the advantages of SLIT over SCIT regard the compliance, which is higher because SLIT does not need to be administered in a medical setting, and cost-effectiveness because there is not the cost of the injections. However, it is important to note that such favourable outcomes take place only if SLIT meets its needs, that is, the administration of high doses is continued regularly for at least 3 consecutive years. In fact, SLIT efficacy is dose-dependent and a sufficient duration is necessary to induce the immunologic changes underlying clinical effectiveness. Recent studies showed that the mechanism of action of SLIT is similar to that demonstrated for SCIT [47] and that when high doses are administered, immunoglobulin G-blocking antibodies, which were not found in SLIT studies employing low doses, are produced in significant amounts and persist also after the discontinuation of treatment [48].

## Competing interests

C. Incorvaia and P. Berto are scientific consultant for Stallergenes, Milan, Italy. S. Masieri has no competing interest.

## Authors' contributions

CI wrote the first draft of the manuscript, SM and SS helped in drafting the manuscript, PB and FF revised the manuscript. All authors have read and approved the final manuscript
